# Psychological Impact of Primary Screening (PIPS) for HPV: a protocol for a cross-sectional evaluation within the NHS cervical screening programme

**DOI:** 10.1136/bmjopen-2016-014356

**Published:** 2016-12-23

**Authors:** Emily McBride, Laura Marlow, Alice S Forster, Sue Moss, Jonathan Myles, Henry Kitchener, Julietta Patnick, Jo Waller

**Affiliations:** 1 Epidemiology and Public Health, Health Behaviour Research Centre, University College London, London, UK; 2 Wolfson Institute of Preventive Medicine, Queen Mary University of London, London, UK; 3 Women's Cancer Centre, Institute of Cancer Sciences, University of Manchester, Manchester, UK; 4 Cancer Epidemiology Unit, University of Oxford, Oxford, UK

**Keywords:** NHS cervical screening programme, Psychological evaluation HPV, HPV primary testing, cervics, Mass screening

## Abstract

**Introduction:**

The NHS Cervical Screening Programme is now using human papillomavirus (HPV) testing as the primary test in six sentinel sites in England, with the intention of rolling this out across the whole of England. Previous research evaluating HPV testing in the cervical screening context suggests that an HPV-positive result may increase anxiety beyond that associated with abnormal cytology, but this has not been explored in the context of primary HPV testing. The main aim of this study is to explore the impact of the HPV primary screening programme on anxiety and distress.

**Methods and analysis:**

A cross-sectional between-groups design (total N ∼ 673) will be employed to assess the psychological impact of different HPV and cytology results at three time points: shortly after receiving the results, and 6 and 12 months later. Women will fall into one of six groups based on their screening results. The primary outcomes will be anxiety and general distress. Secondary outcomes will include understanding of screening results, perceived risk of cervical cancer, psychosexual functioning, intention to attend future screening and knowledge of HPV. General linear modelling will be used to test for differences between groups and changes over the three time points.

**Ethics and dissemination:**

Health Research Authority approval was received on 26 September 2016. Ethical approval was received from London- Surrey Borders NHS Research Ethics Committee on 30 August 2016. Section 251 approval was received from the Confidentiality Advisory Group on 24 August 2016. Results will be disseminated via peer-reviewed publication and presentation at national and international conferences.

**Strengths and limitations of this study**

- This will be the first study to evaluate the psychological aspects of human papillomavirus (HPV) primary testing in routine cervical screening in England.
- This psychological evaluation will complement epidemiological and cost-effectiveness evaluations of HPV primary testing within the National Health Service Cervical Screening Programme (NHSCSP).
- The findings will be very timely, given that the UK Department of Health has recently announced its intention to roll out HPV primary testing nationally. The results of this study will most likely directly inform NHSCSP patient invitation letters, results letters and information materials.
- A cross-sectional between-groups design will be employed, which limits inferences of causality between outcomes. However, this design will allow for an overview of women's responses to receiving different HPV/cytology test results in practice, and follows the same design as adopted in previous psychological evaluations within the NHSCSP.
- Self-selection bias and an anticipated low response rate is likely to generate a sample that is not wholly representative of NHSCSP participants.

## Introduction

In England, the National Health Service Cervical Screening Programme (NHSCSP) aims to prevent cervical cancer by detecting and treating precancerous cervical abnormalities. In recent years, the programme, which has historically used cytology to identify abnormalities in exfoliated cells, has evolved to incorporate the use of human papillomavirus (HPV) DNA testing. In 2010, HPV DNA testing was adopted as a means of triaging borderline and low grade cytology results, and as a test of cure following treatment of cervical intraepithelial neoplasia (CIN).

HPV is a highly prevalent sexually transmitted infection; high-risk types are now accepted to be the main aetiological factor in the development of cervical cancer.^1^ Evidence suggests that using high-risk HPV (hrHPV) DNA testing as the primary test in cervical screening is more sensitive for detecting cervical intraepithelial neoplasia (CIN2 or worse) and may be more cost-effective,^2–5^ although not all studies have found this to be the case.^6^ Also, given that the HPV vaccine was introduced into UK schools in 2008, HPV primary testing may be the most appropriate option for vaccinated cohorts entering the cervical screening programme.^7^ ^8^ In the UK, shifting to a HPV primary testing algorithm would mean that samples taken from women attending cervical screening would first be tested for hrHPV, and cytology would only be carried out on the residual samples of women who were HPV positive. Women who were HPV negative would return to routine recall in 3 or 5 years, while those who were HPV positive would be managed according to their HPV and cytology results. In line with HPV triage methods, women testing positive for hrHPV with abnormal cytology would be referred immediately for colposcopy. However, a key difference of HPV primary testing, relative to the current algorithm, is that it would generate a new group of women with normal cytology and hrHPV-positive results, with these women being recalled for repeat HPV testing at 12 months.

HPV testing has a high negative predictive value, which means that there is the possibility to reassure women who are concerned or anxious about developing cervical cancer and, potentially, to increase the interval between screening tests. Since 2013, the NHSCSP has been using primary HPV testing across six sites in England, and the Department of Health has recently announced its intention to roll this out nationally.^9^ A full description of the primary HPV screening algorithm can be found on the Public Health England website.^10^

The evidence is mixed regarding whether HPV testing in the context of cervical screening is associated with adverse psychological effects. A cross-sectional evaluation of HPV triage in the NHSCSP found temporary adverse psychological effects, whereby increased anxiety, distress and concern were present shortly after women received HPV-positive results, but not at 6 months follow-up.^11^ ^12^ This is in line with qualitative research, which suggested that communication of HPV-positive results may lead to feelings of anxiety, stigma, stress and concern about sexual relationships.^13^ However, a large randomised controlled trial which considered differences in anxiety and distress between women receiving cytology results alone and women also receiving HPV results indicated no overall differences between the groups. The study did find, however, that among women whose HPV results were revealed to them, anxiety and distress were higher in those who received HPV-positive results relative to HPV-negative results.^4^

Thus, although previous research has suggested a trend towards increased anxiety and distress associated with HPV-positive results, the psychological impact is not clear in the context of HPV primary testing, where communication of HPV results to all women entering the programme will be routine and there will be far greater numbers of women receiving hrHPV-positive results compared with HPV triage for low-grade and borderline cytology. Previous research has indicated poor knowledge and understanding of the link between HPV and cervical cancer, and between HPV and sexual activity, among women in the UK.^14–18^ Therefore, if the meaning of HPV results and cancer risk are not well understood, this has the potential to induce unnecessary anxiety. This is particularly relevant for women who are told that they are HPV positive with normal cytology results given that, under the new algorithm, they will be aware that they have hrHPV but there will be no further clinical investigation for 12 months. The likelihood of this subgroup developing cervical cancer in the interim period is extremely low. However, it is possible that women may still feel anxious and/or distressed. Anxiety and distress may be accentuated if women do not fully understand the meaning of these test results. In the light of the high prevalence of HPV, especially in younger women (under 30 years),^19^ it is expected that a large number of women will fall into this new 12-month recall category. In order for the NHSCSP to achieve the sensitivity gains of switching to HPV primary testing, it is important that women in this group attend their recall appointment at 12 months without experiencing significant anxiety in the interim.

### Rationale for the study

With changes to the protocol for screening and follow-up, it is important that psychological factors are evaluated to help determine the information needs and support required for women engaging in HPV primary testing. Information materials for HPV primary testing have already been developed by NHSCSP.^20^ However, it is unclear whether these are sufficient to ensure that women have a good understanding of their screening results and their own cervical cancer risk.

In line with a previous psychological evaluation of HPV triage within the NHSCSP,^11^ ^12^ our primary aim is to consider the impact of this new cervical screening algorithm on anxiety and distress.

Epidemiological and cost-effectiveness analyses of HPV primary testing are already under way. This study protocol is for an evaluation of the psychological aspects of introducing primary HPV testing into the NHSCSP.

## Methods and analysis

### Design

A cross-sectional between-groups design will be employed to assess women at baseline (shortly after receiving their screening result), 6 months postscreening result and 12 months postscreening result.

### Participants and eligibility

Participants will include women aged 25–64 years who have taken part in the NHSCSP in one of five sites where HPV primary testing has been introduced: North London, Sheffield, Norfolk and Norwich, Liverpool and Manchester NHS Trusts.

Eligible women will include those who have received test results within the recruitment period at each NHS site (∼12 weeks recruitment at each).

We will recruit three groups of women following their first HPV test, including those who test negative for HPV, those who are HPV positive with normal cytology and those who are HPV positive with abnormal cytology (groups 1 to 3 in table 1). In addition, we will recruit two groups of women who had initially tested positive for HPV with normal cytology, and who have recently attended their 12-month follow-up appointment, including women who have persistent HPV, and those who tested HPV negative at the recent test (groups 4 and 5 in table 1). We will also recruit a control group of women who have been screened using cytology only and have received a normal result (the participating sites have not yet introduced HPV primary screening for all women). This means there will be a total of six possible combinations of HPV and cytology results for eligibility in this study (six recruitment groups). See table 1 for an overview.

**Table 1:** HPV and cytology results for the six groups included in the study

	HPV result	Cytology result
Group 1	Negative	Not tested
Group 2	Positive	Normal
Group 3	Positive	Abnormal
Group 4*	Persistent positive at 12 months	Normal
Group 5*	Negative at 12 months	None
Group 6 (control)	None	Normal

*Women in groups 4 and 5 will all have tested HPV positive with normal cytology at their first screen and will be recruited to the study after their 12-month follow-up test.

HPV, human papillomavirus.

### Procedures

Eligible patients will be identified by members of staff in cytology departments in NHS laboratories at each of the participating sites. University College London (UCL) will communicate numbers needed in each group to each laboratory as the study progresses, proportionate to the numbers of results processed at each laboratory.

NHS staff will allocate each potential participant a unique identity number, and link this to the patient's name and address. Section 251 approval has been granted to the NHS to upload this information to a secure printing and mailing company (CFH Docmail) for the purposes of contacting participants. Docmail is contractually bound to comply with the Data Protection Act (1998) and securely destroy these data within 30 days of receipt.

Potential participants will be mailed invitation packs to their home address. Invitation packs will include an invitation letter, participant information sheet, consent form and a baseline questionnaire booklet. If participants have not returned the questionnaire after 3 weeks, Docmail will send a reminder pack containing the same documents. Those who opt to take part can do so by returning a completed consent form and questionnaire booklet to UCL.

Participants will also be mailed postal questionnaire packs at 6 and 12 months follow-up. Again, they will be sent a reminder pack containing a reminder letter and another copy of the questionnaire 3 weeks later.

Data from NHS clinical records:

Patient age, index of multiple deprivation score (derived from postcode), date of most recent cervical screen, date of last (previous) cervical screen, number of previous cervical screens and test results will be transferred as population data to UCL from each NHS site for all potential participants approached, with the exception of patients who have opted out. These data will contain no identifiable information; data will be pseudonymised using unique identity numbers. These additional data sets will include survey non-responders to allow for examination of response biases in relation to demographic and screening factors.

### Primary outcomes

State anxiety, measured by the State Trait Anxiety Inventory (STAI-6),^21^ and general distress, measured by the General Health Questionnaire (GHQ-12),^22^ will be the primary outcome measures.

HPV and cytology screening results (groups 1 to 6 as outlined in table 1) will be the independent variable for primary analyses. See table 2 for an overview of primary outcome measures.

**Table 2:** Primary outcome measures

Measure	Description	Source
State-trait anxiety	The state-trait anxiety inventory short-form (S-STAI-6) is a six-item validated questionnaire used to measure state-trait anxiety.^21^	Self-reported by participant in questionnaire.
General distress	The General Health Questionnaire (GHQ-12) is a 12-item validated questionnaire used to measure general distress.^22^	Self-reported by participant in questionnaire.
Test results (HPV and cytology)	HPV and cytology screening results will be communicated to UCL from participating laboratories at NHS sites. Participants will receive one of six possible standardised results (see eligibility criteria for breakdown of groups). Screening result will act as the independent variable for primary analyses.	Communicated to researchers at UCL from NHS clinical records.

HPV, human papillomavirus; NHS, National Health Service; UCL, University College London.

### Secondary outcomes

Understanding of screening results, knowledge of HPV,^23^ perceived risk of developing cervical cancer, concern about screening result, psychosexual functioning^24^ and intention to engage in future screening will act as secondary outcome measures. Health-related quality of life^25^ will also be collected; however, it will be used by Public Health England as part of the health economic evaluation, and will not form part of this psychological evaluation. See table 3 for an overview of secondary outcome measures.

**Table 3:** Secondary outcome measures

Measure	Description	Source
Understanding of results	Understanding of screening results will be measured via a scale developed for this study, which consists of six questions considering perceived meaning of results and cervical screening information sources.Participants will be asked: What do you think your screening result means for your current health?—I have/am likely to have/am unlikely to have/am very unlikely to have/definitely do not have cervical cancer, or I do not know. Can you remember what your screening result was?—HPV and cytology results indicated separately via prompted responses. How confident are you that you understand the meaning of your screening result?—five-point Likert scale ranging from ‘not at all confident’ to ‘very confident’. When you were invited for your recent screening test, how much of the information did you read?—six-point Likert scale ranging from ‘none’ to ‘all of it’, or ‘cannot remember’. Did you look for any extra information about the screening test or your results?—Yes/no/cannot remember. Do you have any unanswered questions about cervical screening or HPV testing?—Presented with free text box. Understanding of results will only be measured at baseline.	Self-reported by participant in questionnaire.
Knowledge of HPV	Knowledge of HPV will be measured using an adapted tool^23^ which asks participants to answer true or false to 10 statements about HPV. This tool has been adapted by only including those questions reflective of the information provided to women in the NHSCSP materials. Participants will also be asked whether they have heard of HPV before today and how they would rate their knowledge on a five-point Likert scale between ‘very poor’ and ‘very good’. Knowledge will only be measured at baseline.	Self-reported by participant in questionnaire.
Perceived risk of cervical cancer	Perceived risk of developing cervical cancer will be assessed by asking participants to answer: ‘Compared with other women the same age as you, do you think your chances of developing cervical cancer in the next 10 years are…?’ Answers will range on a five-point Likert scale from ‘much below average’ to ‘much above average’. This is an adapted scale from Maissi *et al*.^11^ ^12^	Self-reported by participant in questionnaire.
Concern	As in Maissi *et al*,^11^ ^12^ concern will be measured by asking (1) how concerned and (2) how reassured do you feel about your recent screening result? Participants will also be asked an additional question: ‘how worried are you about getting cervical cancer in the next 10 years?’	Self-reported by participant in questionnaire.
Intention to attend future screening	Participants will be asked one question: ‘will you go for cervical screening next time you are invited?’ Answers will be indicated on a five-point Likert scale ranging between ‘yes, definitely’ and ‘definitely not’.	Self-reported by participant in questionnaire.
Psychosexual functioning	The Psychosocial Effects of Abnormal Pap Smears Questionnaire short-form (PEAPS-Q-5) is a five-item validated questionnaire used to measure distress experienced by women undergoing follow-up investigation after an abnormal Pap smear result.^24^ Not all participants will have received abnormal test results; therefore, we slightly adapted this scale by inserting a ‘not applicable’ option after each question.	Self-reported by participants with HPV+results in questionnaire.
Health-related quality of life	The Health-Related Quality of Life Questionnaire short-form (EQ-5D) is a 5-item validated tool used to assess five dimensions related to quality of life: mobility, self-care, usual activities, pain/discomfort and anxiety/depression.^25^ This will be collected by UCL but analysed and reported as part of the PHE health-economic evaluation.	Self-reported by participant in questionnaire.

HPV, human papillomavirus; NHSCSP, National Health Service Cervical Screening Programme; PHE, Public Health England; UCL, University College London.

### Descriptive measures

Age, ethnicity, marital status, index of multiple deprivation (a measure of deprivation linked to an individual's residential postcode), education level, NHS site, previous screening history and HPV vaccine status will be collected for descriptive information and as potential control factors. See table 4 for an overview.

**Table 4:** Descriptive outcome measures

Measure	Description	Source
Demographics	Ethnicity, educational attainment, employment and marital status will be assessed via questionnaire. Age of participant will be communicated to UCL from participating laboratories at pilot sites. This will be measured at baseline.	Self-reported in questionnaire. Age at baseline, communicated to UCL from NHS clinical records.
Index of multiple deprivation	Index of Multiple Deprivation score (IMD) will be assigned to participants by laboratories and communicated to UCL. The IMD is a measure of area level deprivation which can be derived from a postcode.^26^ It takes into account: income deprivation; employment deprivation; education, skills and training deprivation; health deprivation and disability; crime; barriers to housing services; and living environment deprivation.	Calculated by the NHS via clinical records and communicated to UCL in score form.
NHS Site	The NHS site where women are screened and receive test results will be recorded via communication from participating laboratories.	Communicated to UCL by NHS site.
Previous screening history	Previous screening history will be communicated to UCL from participating laboratories. This information will include date of last screening and number of previous screenings.	Communicated to UCL from NHS clinical records.
HPV vaccine status	Participants will be asked to indicate whether they have received the HPV vaccine and how many doses they had. This will be measured at baseline.	Self-reported by participant in questionnaire.

HPV, human papillomavirus; NHS, National Health Service, UCL, University College London.

### Sample size

The study has been powered to detect a small-to-medium between-group difference (f=0.14) in anxiety (as measured by the STAI-6).^11^ On the basis of previous studies,^11^ ^12^ we expect anxiety scores across groups to be in the range of 36–40, with an SD of 12. With an α of 0.05, a sample size of 673 will give us 80% power to detect a between-group difference in anxiety.

Assuming an initial response rate of 35%, with 75% of initial responders returning a second questionnaire at 6-month follow-up, and 75% of responders at 6 months completing a third questionnaire at 12 months, we plan to approach 3415 participants to achieve the target sample size. Response rate will be monitored as the study progresses so that the number of women approached at baseline can be adjusted if the response rate is higher or lower than expected (within our funding constraints).

### Data analyses and statistics

Data will be coded and analysed using SPSS, R and Stata. An α level of p<0.05 will be used throughout.

Preliminary analyses (analysis of variance (ANOVAs) and χ²) will be conducted to explore descriptive statistics and to identify significant group differences, as potential control measures, for age, index of multiple deprivation, pilot site, screening result, previous screening history, HPV vaccine status, marital status, ethnicity and educational attainment. Primary analyses will comprise between-groups ANOVAs to explore whether anxiety and general distress differ between screening result groups shortly after initial presentation of screening result (baseline).

Mixed ANOVAs will be conducted to explore whether differences in anxiety and general distress are observed between screening result groups over time (baseline, 6 months and 12 months).

General linear modelling will be conducted to consider whether understanding of screening results, knowledge of HPV, perceived risk of developing cervical cancer, concern about screening result, psychosexual functioning and intention to engage in future screening differ between screening result groups for secondary analyses.

Health-related quality of life data will be analysed in the health economic cost-effectiveness evaluation (not as part of this psychological evaluation).

Post hoc comparisons will be conducted where appropriate and effect sizes will be calculated.

## Discussion

This psychological evaluation of HPV primary testing within the NHSCSP will provide evidence about the psychological consequences of testing positive for HPV in this context and is expected to show that the negative consequences are minimal and short-lived, as has been found when HPV testing is used to triage women.^11^ ^12^ The findings should help identify any unmet information needs of women taking part in the programme. If adverse psychological effects are observed, the results will help to inform the development of materials and/or procedures aimed at ensuring clarity in the meaning of test results and reducing psychological burden. Given that a ministerial announcement has now been made, stating that HPV primary screening is to be rolled out across England and incorporated into routine NHS practice,^9^ the study findings are likely to directly inform finalised NHSCSP test invitation letters, result letters and accompanying HPV information materials. They may also help inform the development of pragmatic interventions (eg, training programmes, written information) for healthcare professionals working in cervical screening, to promote effective communication and address common concerns for women undergoing HPV primary screening.

In addition, we will be gathering certain population-level data from NHS databases on all women approached to take part in this study, including index of multiple deprivation score, age, test results and cervical screening history. From this information, along with questionnaire data collected from study participants, we will explore predictive relationships between outcomes. This may help identify certain groups of women likely to need additional support. For example, previous research has suggested that younger age and lower understanding of the meaning of test results predicts higher anxiety among women who are HPV positive.^11^ In the context of HPV primary testing, if results indicate similar patterns, this may highlight groups of women requiring additional information and/or interventions to minimise any adverse psychological effects.

We will also be asking women to self-report their test results in addition to collecting the same information from NHS clinical databases. This will allow for direct comparison between women's understanding of their test results and objective clinical data. From these comparisons, we will be able to explore whether women accurately interpret the meaning of their HPV and cytology test results.

## Dissemination

We plan to publish the results of this study in two peer-reviewed journal articles. In the first paper, we will report between-group differences at baseline for all outcomes. In the second paper, we will report outcomes which are relevant to analyses over time (6 and 12 month follow-up) and explore predictive relationships. Results will also be disseminated through presentations at national and international conferences and will be communicated to the NHSCSP and relevant third sector organisations, such as Jo's Cervical Cancer Trust.
